# Cerebral folate deficiency in two siblings caused by biallelic variants including a novel mutation of *FOLR1* gene: Intrafamilial heterogeneity following early treatment and the role of ketogenic diet

**DOI:** 10.1002/jmd2.12206

**Published:** 2021-06-04

**Authors:** Maria T. Papadopoulou, Efterpi Dalpa, Michalis Portokalas, Irene Katsanika, Katerina Tirothoulaki, Martha Spilioti, Spyros Gerou, Barbara Plecko, Athanasios E. Evangeliou

**Affiliations:** ^1^ Division of Child Neurology and Inherited Metabolic Diseases, 4th Department of Pediatrics Aristotle University of Thessaloniki, ‘Papageorgiou’ General Hospital Thessaloniki Greece; ^2^ Diet & Nutrition Department ‘Papageorgiou’ General Hospital Thessaloniki Greece; ^3^ 1st Department of Neurology Aristotle University of Thessaloniki, ‘AHEPA’ Hospital Thessaloniki Greece; ^4^ Analysis Medical SA Thessaloniki Greece; ^5^ Department of Pediatrics and Adolescent Medicine, Division of General Pediatrics Medical University of Graz Graz Austria

**Keywords:** cerebral folate deficiency, epileptic spasms, folinic acid, FORL1, FRa, ketogenic diet

## Abstract

Mutations in the *FOLR1* gene, encoding for the folate alpha receptor (FRa), represent a rare recessive genetic cause of cerebral folate deficiency (CFD), a potentially reversible neurometabolic condition. Patients typically present with developmental delay, seizures, abnormal movements, and delayed myelination. We hereby expand the phenotypic and genotypic spectrum of the disease with the report of the first two Greek siblings that were found compound heterozygous for one known *FOLR1* gene mutation (p.Cys65Trp) and a mutation (p.Trp143Arg) that has not yet been reported in the literature (class 3 variant according to ASHG classification). A distinguishing feature of the older sibling is the manifestation of drug‐resistant epileptic spasms beyond infancy. These had a relatively good response to a ketogenic diet, as an additional treatment to topiramate and valproate. A further clinical improvement was observed when folinic acid was combined with the above treatment. While a response to folinic acid is well established in the disorder, the efficacy of its combination with the ketogenic diet needs further evaluation, but we suggest considering it early in the course of drug resistant epilepsy in the setting of CFD. The younger sibling was diagnosed and treated with folinic acid at an early‐symptomatic stage. Both patients had moderately low age‐related CSF 5‐methyltetrahydrofolate levels at diagnosis with the older sibling (that was already treated at base line collection) averaging 19 nmol/L (normal range: 44‐122 nmol/L) and the younger one 49 nmol/L (normal range 63‐122 nmol/L). These levels were restored to normal limits after folinic supplementation.

**SYNOPSIS**

Ketogenic diet could be considered in cases of drug resistant epilepsy related to CFD‐FOLR1 disorder as an add‐on treatment to antiepileptic medication and/or to folinic acid. Early folinic acid administration represents a disease‐modifying treatment. Lowering of MTHF in CSF is age‐specific and may be mild to moderate, especially in younger patients and at early stages of the disease.

## 1 INTRODUCTION

Folic acid is a water soluble B‐complex vitamin that is reduced to several metabolites (dihydrofolate, tetrahydrofolate, folinic acid, 10‐formyl‐tetrahydrofolate, 5,10‐methylene‐tetrahydrofolate, 5‐methyl‐tetrahydrofolate, etc) in the human body. All of these play a key role as cofactors or coenzymes in various metabolic processes involving DNA repair, gene expression, amino‐acid/neurotransmitter metabolism, pyrine and pyrimidine biosynthesis and choline formation, that are essential for normal myelination.^1^ 5‐methyltetrahydrofolate (5‐MTHF) is the main transport folate form, mediated by several transport systems, such as the reduced folate carrier (RFC) and the proton‐coupled folate transporter (PCFT). 5‐MTHF also crosses the blood‐CSF‐barrier through binding with the high 5‐MTHF affinity folate receptors a (FRa) of the choroid epithelial cells and serves as the main folate source of the central nervous system.^2^ Cerebral folate deficiency (CFD) was first described less than 20 years ago as a neurometabolic condition with low CSF 5‐methyltetrahydrofolate (5‐MTHF) in the setting of normal systemic folate levels.^3^ CFD is a potentially reversible condition as symptoms are described to be responsive to folinic acid administration.^4^

CFD differential diagnosis includes several primary and secondary conditions that could interfere with transport, metabolism, and recycling of 5‐MTHF in the central nervous system.^5^ Primary conditions include rare inborn errors of folate transport and metabolism, such as deficiency of methylenetetrahydrofolate reductase, dihydrofolate reductase, methenyl‐tetrahydrofolate synthase, and folate receptor alpha. Among the most frequent secondary CFD causes are Kearnes‐Sayre syndrome and other mitochondrial disorders, serine deficiency, pyridoxine‐dependent epilepsy and folate receptor‐a (FRa) antibodies.^1^, ^5^ Primary causes are related to more severe CFD, while mild to moderate CFD has been reported in >30 neurological and genetic conditions, including Rett syndrome, Aicardi‐Goutières syndrome, CNS hypoxia, epileptic syndromes, but also antiepileptic and other drug administration.^6^

## 2 CASE PRESENTATION

**FIGURE 1:**
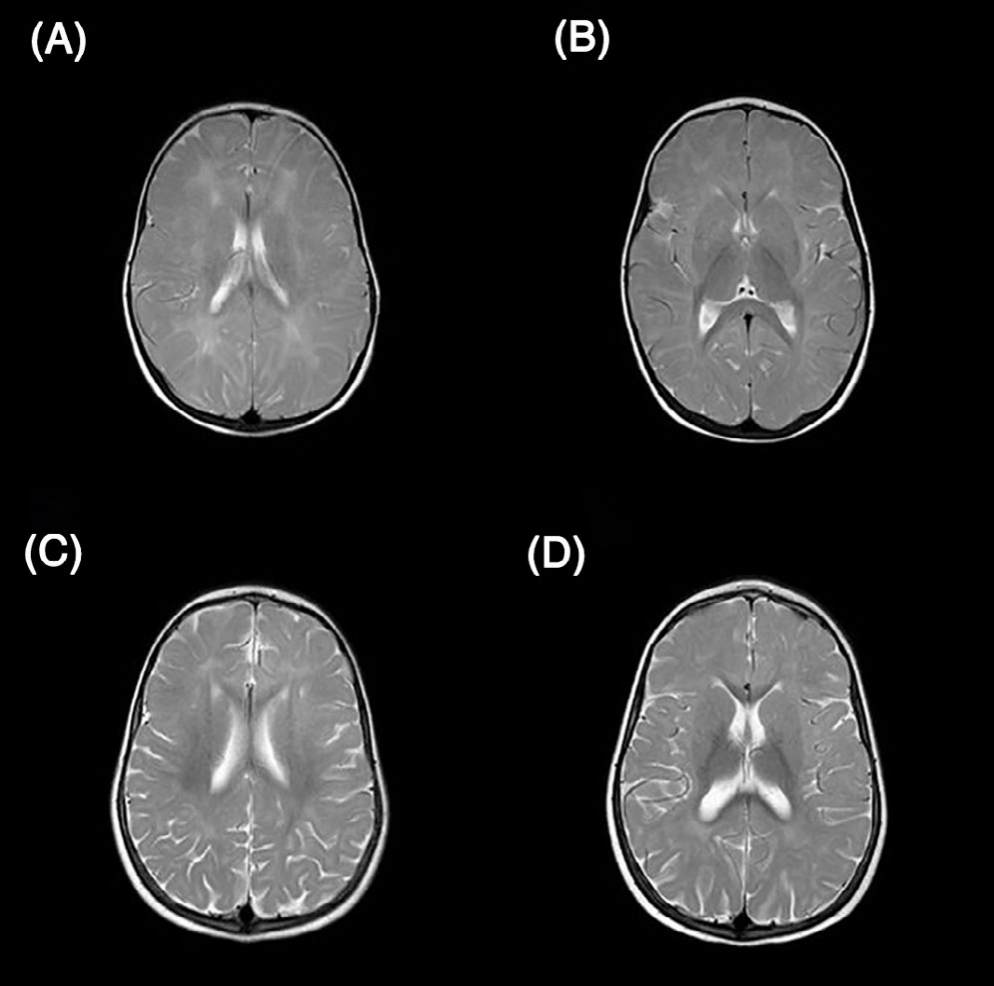
T2 brain MRI of sibling #1 showed (Α and Β) delay in myelination of subcortical and periventricular white matter at the age of 19 months with normal CNS structure, (C and D) persistence of the axonal damage at the age of 3 years after 6 months of folinic acid administration, accompanied by clinical improvement

**FIGURE 2:**
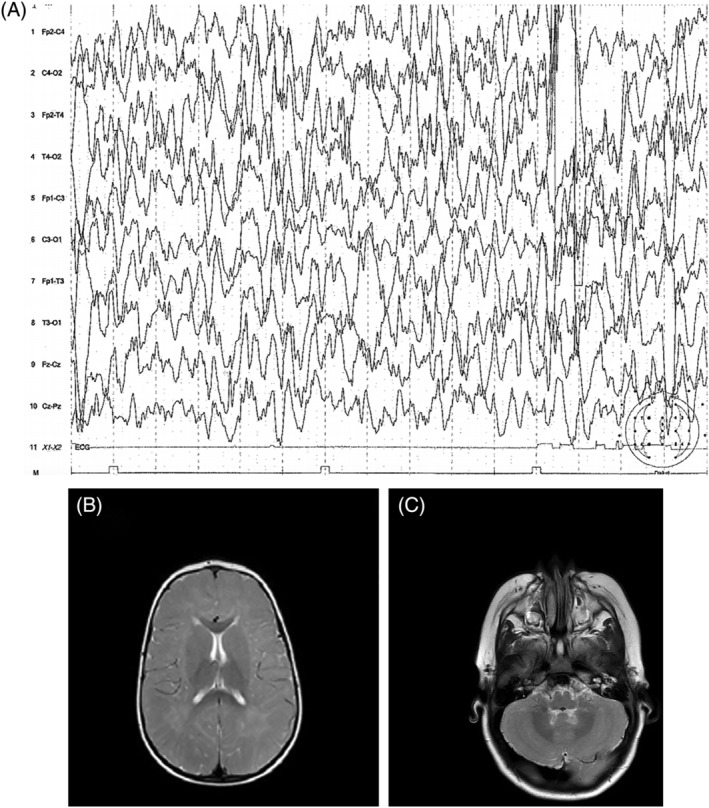
A, Hypsarrhythmic‐like interictal EEG activity of the older sibling at the age of 22 months, marked by theta/delta waves with poor spatiotemporal organization and interrupted by frequent multifocal spikes and intermittent bouts of hypsarrhythmia. B and C, T2 brain MRI of sibling #2 at the age of 18 months, after a few months of per os folinic acid, showing a similar pattern of delayed myelination as his sister, although he clinically presented only mild developmental delay and ataxia

A 19 months old female patient was referred to our department because of developmental delay, hypotonia and mild ataxia. She had an unremarkable family, perinatal and personal history with normal developmental milestones until the age of 11 months and an otherwise normal physical examination (Head Circumference [HC]: 45.5 cm, 10th‐25th percentile). The initial laboratory and metabolic work‐up was normal, while a brain MRI showed delay in myelination (Figure 1A,B). Important developmental regression at the age of 2 years (loss of speech and imitation, aggravation of ataxia, loss of standing position and fine motor skills as independent eating, appearance of autistic behavior) was followed 2 months later by onset of several daily clusters of epileptic spasms and abnormal EEG background activity (Figure 2A). The HC was still in the normal range (47 cm, 10th to 25th percentile).

The epileptic spasms were drug‐resistant, and the patient also presented other seizure types within few months, only partially responding to antiepileptic treatment (Table 1/Supplementary file 1). Ketogenic diet (KD), initiated 3 months after seizure onset as an add‐on treatment to valproate and topiramate, resulted in a further 50% reduction of seizure frequency (approximately 10 episodes per day reduced to 5 per day) and severity with a significant improvement in the patient's daily life. Meanwhile, the repeat of the metabolic work‐up with CSF organic acids, amino acids, and neurotransmitters (without 5‐MTHF) was nonconclusive. Folic acid in plasma was measured at lower normal levels (4.5 ng/mL) and per os supplementation with folic acid at a daily dose of 1 mg had a beneficial effect on patient's development, according to the parents. After suspicion of CFD due to delayed myelination, ataxia, and epilepsy, folinic acid was also administered at a daily dose of 3 mg/kg/d per os with clinical improvement in social interaction and motor skills. Whole Exome Sequencing revealed two compound heterozygous mutations in the *FOLR1* gene, ΝM_016725: c.195C > G (p.Cys65Trp) and c.427 T > A (p.Trp143Arg). Both parents were carriers of a single variant and the patient's older asymptomatic sister had only inherited the paternal mutation. Because of the low CSF age‐related 5‐MTHF levels (19 nmol/L, normal range 44‐122 nmol/L^7^) and the incomplete clinical response to per os folinic acid administration at a dose of 3‐5 mg/kg/d for 3 months, along with, 3 day regimens of IV calcium folinate (10 mg/kg/day) were administered weekly under careful laboratory and clinical monitoring. The patient showed immediate clinical improvement with diminution of seizures (ca. 1 per day) and regained walking with support, imitating, eye contact and some verbal communication within 2 months. No acute adverse effects were reported during IV administration but mild leucopenia (~3.500‐4.500 leukocytes, neutrophils 900‐1100) was probably linked to the treatment, after a negative extended etiological work‐up. As no effect on epilepsy was observed by any other intervention apart from KD and folinic acid, the attempt to slowly discontinue all the antiepileptic drugs was successful, with no change in seizure frequency, type, and duration. After 4 months of IV calcium folinate administration, the patient was slowly but constantly progressing (regained walking with support, use of some words), the seizures were stabilized (<1 per day), the brain MRI at the age of 2.5 years confirmed hypo‐myelination with no further findings (Figure 1C,D) and CSF 5‐MTHF levels were augmented to 52 nmol/L.

**TABLE 1:** Summary of patients' main characteristics and follow‐up

	Age (last follow‐up/symptom onset)	Symptoms/findings	Epilepsy (seizure type/age of onset)	Responsiveness to antiepileptic treatment before	CSF levels (at diagnosis)	Treatment at last follow‐up	Follow‐up
Sibling #1	3.5 years/15 months	Hypotonia, ataxic gait, developmental regression, autistic features, hypomyelination (brain MRI)	Mainly epileptic spasms, absences, tonic, GTCS/26 months	VGB, cortico‐steroids, VPA, TPM (resistant) Folic acid, B6 (transient partial response) KD (>50% reduction)	5‐MTHF: 19 nmol/L, (normal range: 44–122 nmol/L) 5HIAA: 172 nmol/L (normal range: 170‐490 nmol/L) HVA: 481 nmol/L (normal rane: 344‐906)	KD (3.5:1) folinic acid per os 6 mg/kg/d + twice weekly IV calcium folinate 10 mg/kg	<1 seizure/day ataxia, mild spasticity slowly regains some dd milestones
Sibling #2	2 years/12 months	Hypotonia, mild dyskinesia, autistic features hypomyelination (brain MRI)	–	–	5‐MTHF: 49 nmol/L (normal range: 63‐122 nmol/L)	Folinic acid per os 6 mg/kg/day	Walks without assistance/uses few words/developmental progress

Abbreviations: 5HIAA, 5 hydroxy indolacetic acid; GTCS, generalized tonic‐clonic seizures; HVA, homovanilic acid; TPM, topiramate; KD, ketogenic diet; VGB, vigabatrin; VPA, valproate.

Compound heterozygous mutations of *FOLR1* gene were also detected in the patient's younger brother; 5‐MTHF levels in CSF were measured at 49 nmol/L at diagnosis (normal range for age: 63‐122 nmol/L^7^). His development was considered within the normal limits until the age of 1 year, although concerns were raised with regards to some involuntary movements and relevant stagnation of development around that age. He was immediately administered per os calcium folinate (initially 2 mg/kg/d). By the age of 18 months, dyskinetic and stereotypic features were apparent but he walked without assistance, followed simple orders, used a few words and had a good nonverbal communication. He never experienced epileptic seizures. His baseline EEG was normal, but his brain MRI at diagnosis had already extended delay in myelination (Figure 2B,C, Table 1). Per os calcium folinate was elevated to 6 mg/kg/d according to clinical response and repeat of CSF 5‐MTHF levels were found within normal limits for this age (71 nmol/L, normal range: 44‐122 nmol/L).

Both siblings have to date a normal growth, normal HC and no abnormal ophthalmological and hearing findings. They are under clinical and biochemical monitoring every 3 months.

## 3 DISCUSSION

Mutations in the *FOLR1* gene (OMIM #613068) encoding for the folate alpha receptor represent a rare cause of CFD with only 23 cases described to date.^5^ This autosomal recessive disorder was first described by Steinfeld et al^8^ in 2009, who reported two German siblings with compound heterozygosity for two nonsense mutations and one Italian patient with a homozygous duplication in *FOLR1*. Impaired transcription and severely diminished FRa expression was biochemically proven for all three patients that shared clinical and neuroimaging features; all three showed some improvement under per os folinic acid administration.^8^

We report the first two siblings in Greece with compound heterozygous missense mutations in *FOLR1*, inherited from their healthy, nonconsanguineous, parents of Greek origin. The paternal mutation c.195C > G (p. Cys65Trp) had previously been described in a homozygous Azerbaijani patient,^9^ but the maternal missense c.427 T > A (p.Trp143Arg) mutation is novel. According to the ASHG classification^10^ the c.427 T > A (p.Trp143Arg) variant has been interpreted as a class 3 variant, a variant of uncertain significance; it should thus be interpreted with caution. However, its pathogenicity is supported by the in silico predictive tools, the phenotype, the low age‐related 5‐MTHF levels in patients' CSF, the compound heterozygosity and the cosegregation in the family. The *FOLR1* gene has been sequenced as part of WES in full (100% coverage for this gene) in all involved individuals and no further mutation has been identified. In addition, this variant is not present in international databases (ClinVar, HGMD, 1000GP, etc).

Grapp et al further studied the effect of p.Cys65Trp mutation and other homozygous or compound heterozygous missense mutations in *FOLR1* of 7 patients. They report that the impairment in folate binding was due to loss of FRa cell surface localization and mistargeting to intracellular compartments, even in cases of a very small decrease in protein expression.^9^ The spectrum of FRa deficiency is still expanding but patients described so far have some common clinical and neuroimaging features.^11^ A symptom‐free period that ranges from 4 months to 4.5 years is probably linked to the intrauterine expression of folate receptor beta (FRβ) that assures brain folate transport during early life.^12^ Symptoms are initially motor anomalies (hypotonia, tremor, ataxia, etc) and speech delay with subsequent developmental regression and seizures. Other clinical features include irritability, spasticity, autistic behavior, acquired (only 1 patient with congenital) microcephaly and polyneuropathy.^5,^ ^9^

Seizure onset is usually after 18 months of age and several seizure types including astatic, tonic, tonic‐clonic and atypical absences have been described, along with recurrent episodes of status epilepticus.^5^ Epilepsy onset with epileptic spasms and hypsarrhythmic‐like pattern is a distinguishing feature of the older sister. This unique clinical presentation, led to the induction of a ketogenic diet. Data on EEG ictal and interictal activity is very limited (slow background activity, multifocal discharges, etc) in existing literature, followed by a lot of uncertainties on whether any drug other than folinic acid is efficient on FRa related seizures.^13^ Ketogenic diet as an add‐on treatment was beneficial for the drug resistant epilepsy of the older sibling, with approximately 50% of seizure reduction, possibly linked to its established benefit in mitochondrial function. Further significant clinical improvement was achieved after a combination of per os and IV calcium folinate was added to the treatment (Supplementary file 1).

Disturbed myelination becomes apparent early in the course of the disease; other usual neuroimaging findings include cerebral and cerebellar atrophy, T2 hyperintensities and low choline/inositol metabolites in MR spectroscopy.^5^, ^9^ Severe delay in myelination was apparent even in the younger, affected brother, just after the appearance of mild symptoms. On the other hand, none of the siblings presented brain atrophy. Although there is evidence that brain atrophy progresses with time in untreated patients,^13^ further investigations are needed to correlate this feature to either early diagnosis and treatment or interindividual differences.

Surprisingly, the untreated younger brother at the age of 12 months had relatively high‐for‐age 5‐MTHF CSF levels in comparison to the levels of his sister at the age of 32 months. The CSF collection and analysis were identical between siblings; the older sister was under treatment with per os calcium folinate at diagnosis. 5‐MTHF levels of younger sibling were also higher compared to previous patients with *FOLR1* deficiency (usually <5 nmol/L). Differences in analytical methods across countries/groups are largely eliminated by compliance to internationally standardized procedures.

Previous reports have proven gradual diminution of 5‐MTHF levels in untreated patients with CFD.^3^, ^14^ Measurement of plasma and CSF 5‐MTHF levels in a cohort of 538 patients illustrated, that profound MTHF deficiency in CSF is more likely associated with monogenic disorders such as *FOLR1* deficiency, while mild to moderate CFD in infancy may be associated with a variety of neurological conditions and may represent a secondary phenomenon.^14^ The moderate decrease of CSF 5‐MTHF of the siblings in our report indicates that the absolute levels of CSF 5‐MTHF per se do not allow discrimination between genetic defects primarily affecting folate metabolism and secondary CFD related to other CNS diseases, especially when the CSF collection is performed at young age and/or an early symptomatic state.

In fact, a clear correlation between genetic‐clinical‐neuroimaging and biochemical findings in *FOLR1* deficiency has not been established and management can be very challenging. Several different regimens of folinic acid supplementation have been proposed. When oral supplementation with 2‐10 mg/kg of folinic acid is not sufficient to achieve clinical improvement, additional intravenous injections of folinic acid weekly may be necessary (we used an adjusted‐upon‐response regimen of IV ~10 mg/kg calcium folinate twice weekly). Other experts also propose IV 50‐100 mg folinic acid once per week and intrathecal administration of folinic acid in selected patients.^15^ Response to folinic acid administration varies significantly between individuals, but folinic acid has been proven beneficial even in adult patients.^16^ The benefit of folinic acid supplementation reported in the case of the older sibling is difficult to be interpreted, as it was administered in combination with ketogenic diet and antiepileptic treatment. Several challenges arise with regards to administration type/dosage/folate form/adverse effects of treatment. Early diagnosis and folinic acid administration remains the management gold standard that can possibly alter the course of the disease, as suggested by familial cases with early and late treatment initiation.

## CONFLICT OF INTEREST

Maria T. Papadopoulou, Efterpi Dalpa, Michalis Portokalas, Irene Katsanika, Katerina Tirothoulaki, Martha Spilioti, Spyros Gerou, Barbara Plecko, and Athanassios Evangeliou declare that they have no conflict of interest.

## AUTHOR CONTRIBUTIONS

Maria T. Papadopoulou, Martha Spilioti, Spyros Gerou, and Athanassios Evangeliou contributed to the planning of this work. Maria T. Papadopoulou, Michalis Portokalas, Katerina Tirothoulaki, and Athanassios Evangeliou were involved in the management of the patients and provided important clinical data. Maria T. Papadopoulou, Efterpi Dalpa, Barbara Plecko, and Athanassios Evangeliou drafted the manuscript. Michalis Portokalas, Irene Katsanika, and Katerina Tirothoulaki carefully consulted patient's files for necessary information. Spyros Gerou supervised the genetic testing and provided important information related to the causative mutations. Efterpi Dalpa, Barbara Plecko, and Martha Spilioti also contributed to the preparation and design of the manuscript by analyzing patient's data, reviewing the literature and providing expert opinion. All authors reviewed the final manuscript and approved its submission.

## ETHICS STATEMENT

All procedures followed were in accordance with the ethical standards of the responsible committee on human experimentation (institutional and national) and with the Helsinki Declaration of 1975, as revised in 2000. Additional parental informed consent was obtained on behalf of the patients for identifying information and is available upon request. All data and images of this report are available from the corresponding author upon request.

## Supporting information


**Appendix** **S1**. Supporting information
