# Supplementary material for: Cerebral folate deficiency in two siblings caused by biallelic variants including a novel mutation of FOLR1 gene: Intrafamilial heterogeneity following early treatment and the role of ketogenic diet
Source: JIMD Rep. 2021 Jun 4;60(1):3–9. doi: 10.1002/jmd2.12206 (PMC8260484; doi:10.1002/jmd2.12206)
Supplement: Supplementary file 1 — Appendix S1. Supporting information [file JMD2-60-3-s001.pdf]

	Treatment initiation	1 week	3weeks	4 weeks	6 weeks	8 weeks	2 months	3 months	4 months	6 months	9 months
Prednisolone mg/kg			5	6	2	0					
Vigabatrin mg/kg	100	150	150	100	0						
B6 mg IM for 5 days			100								
Topiramate mg/kg						5	8	8	8	5	0
Ketogenic Diet 3:1 / 3,75:1								100	100	100	100
Valproate mg/kg					30	40	40	40	30	30	0
Calcium folinate per os									3	5	5
Calcium folinate IV										10	10
Siezures per day	15	15	10	10	15	10	10	5	5	1	1

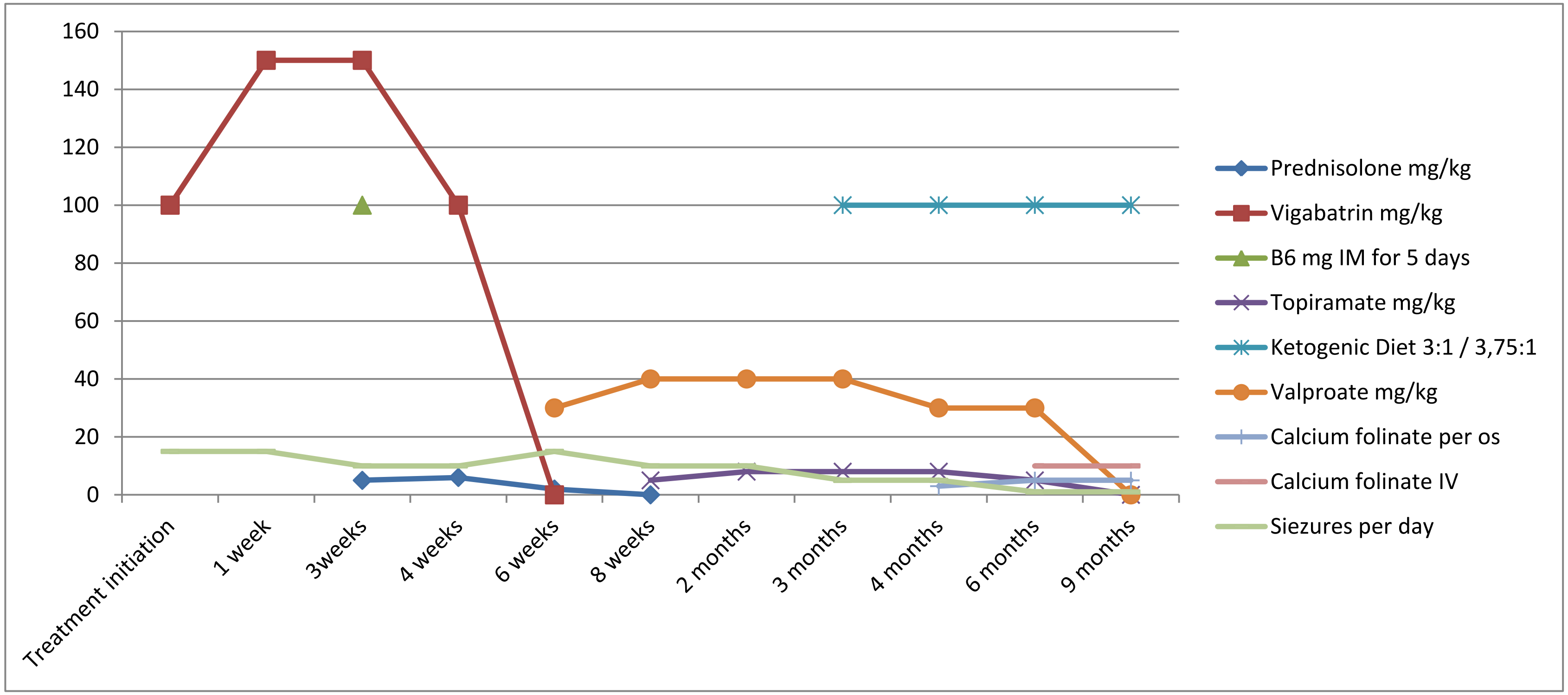

SCHEMA 1: Sibling #1 Treatment dosage and duration.
